# Comparative Analysis of the Clinical Presentation of Individuals Who Test Positive or Negative for SARS-CoV-2: Results from a Test Street Study

**DOI:** 10.3390/v16071031

**Published:** 2024-06-26

**Authors:** Pantea Kiani, Pauline A. Hendriksen, Andy J. Kim, Johan Garssen, Joris C. Verster

**Affiliations:** 1 Division of Pharmacology, Utrecht Institute for Pharmaceutical Sciences, Utrecht University, 3584CG Utrecht, The Netherlands; p.kiani@uu.nl (P.K.); p.a.hendriksen@students.uu.nl (P.A.H.); j.garssen@uu.nl (J.G.); 2 Department of Psychology and Neuroscience, Dalhousie University, 1355 Oxford Str., Halifax, NS B3H 4R2, Canada; andy.kim@dal.ca; 3 Danone, Global Research & Innovation Center, 3584CT Utrecht, The Netherlands; 4 Centre for Mental Health and Brain Sciences, Swinburne University, Melbourne, VIC 3122, Australia

**Keywords:** COVID-19, SARS-CoV-2, symptoms, loss of smell, predictors, mood, quality of life, sleep quality

## Abstract

The common cold, the flu, and the 2019 coronavirus disease (COVID-19) have many symptoms in common. As such, without testing for severe-acute-respiratory-syndrome-related coronavirus 2 (SARS-CoV-2), it is difficult to conclude whether or not one is infected with SARS-CoV-2. The aim of the current study was to compare the presence and severity of COVID-19-related symptoms among those who tested positive or negative for the beta variant of SARS-CoV-2 (B.1.351) and identify the clinical presentation with the greatest likelihood of testing positive for SARS-CoV-2. *n* = 925 individuals that were tested for SARS-CoV-2 at Dutch mass testing sites (i.e., test streets) were invited to complete a short online survey. The presence and severity of 17 COVID-19-related symptoms were assessed. In addition, mood, health correlates, and quality of life were assessed for the week before the test. Of the sample, *n* = 88 tested positive and *n* = 837 tested negative for SARS-CoV-2. Individuals who tested positive for SARS-CoV-2 reported experiencing a significantly greater number, as well as greater overall symptom severity, compared to individuals who tested negative for SARS-CoV-2. A binary logistic regression analysis revealed that increased severity levels of congestion, coughing, shivering, or loss of smell were associated with an increase in the odds of testing positive for SARS-CoV-2, whereas an increase in the severity levels of runny nose, sore throat, or fatigue were associated with an increase in the odds of testing negative for SARS-CoV-2. No significant differences in mood or health correlates were found between those who tested positive or negative for SARS-CoV-2, except for a significantly higher stress score among those who tested negative for SARS-CoV-2. In conclusion, individuals that tested positive for SARS-CoV-2 experienced a significantly greater number and more severe COVID-19-related symptoms compared to those who tested negative for SARS-CoV-2. Experiencing shivering and loss of smell may be the best indicators for increased likelihood of testing positive for SARS-CoV-2.

## 1. Introduction

The global outbreak of coronavirus disease (COVID-19), caused by the severe acute respiratory syndrome coronavirus 2 (SARS-CoV-2), has presented unparalleled challenges to the healthcare community worldwide. Among these, the development and implementation of effective diagnostic strategies have been a major strategy to control the virus’s spread. The Dutch public health response, adhering to international guidelines from June 2020, advocated for widespread testing among individuals displaying mild COVID-19 symptoms or those recently exposed to confirmed cases [1]. This policy, while theoretically sound, encountered significant practical difficulties, particularly due to the non-specific nature of COVID-19 symptomatology, which closely mirrors that of other respiratory infections such as influenza and the common cold (see Table 1) [2,3,4].

Commonly reported symptoms in all three conditions include coughing, runny nose, sore throat, headache, muscle pain, and fever (with a body temperature ≥ 38 Celsius). While the flu and COVID-19 generally exhibit higher symptom severity ratings compared to the common cold, the commonly reported symptoms can be present in all three conditions, with severity ratings ranging from absent, mild, or moderate to severe. More distinctive symptoms of COVID-19, such as cyanosis of the lips or sudden onset of confusion, though clinically significant, are infrequently observed, limiting their diagnostic utility. Thus, based on the clinical presentation of symptoms, it is difficult for individuals to predict whether or not they were infected with SARS-CoV-2. As a result, studies revealed that the large majority of participants tested negative for SARS-CoV-2 [5,6,7]. This approach posed a significant burden on healthcare—both financially and in terms of personnel usage.

Ideally, individuals should be able to make an informed decision about the likelihood of testing positive for SARS-CoV-2 based on their symptoms. Therefore, this study aimed to identify the clinical presentation with the greatest likelihood of testing positive for SARS-CoV-2. To this extent, the presence and severity of COVID-19-related symptoms among individuals that were tested for SARS-CoV-2 in Dutch test streets were evaluated. Participants were grouped based on testing positive or negative for SARS-CoV-2. For both groups, the presence and severity of COVID-19-related symptoms were recorded at the moment of testing, along with retrospective assessments of mood and health correlates for the week before testing. The study’s objective was to determine the extent to which the clinical presentation differed between individuals who tested positive or negative for SARS-CoV-2.

## 2. Materials and Methods

The Corona Test Street Study (COTEST) was conducted among Dutch adults (18 years and older) who completed a SARS-CoV-2 rapid antigen test between December 2020 and June 2021. The Ethics Committee of the Faculty of Social and Behavioral Sciences of Utrecht University approved the study (approval code: FETC17-061) and all participants provided electronic informed consent.

**Figure 1:**
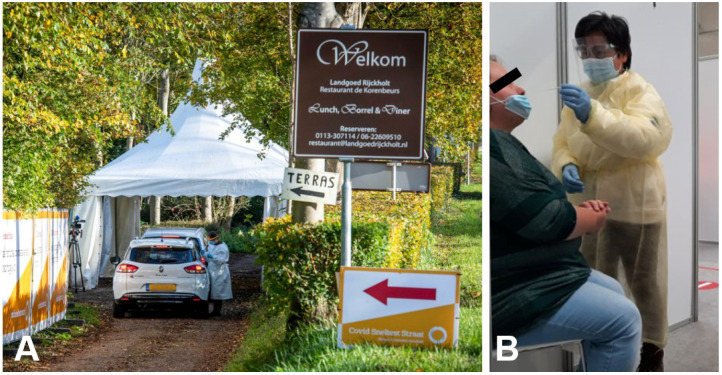
Illustration of a COVID-19 test street. Participants who entered by car were tested while remaining in their car (**A**), while those who came by foot or bicycle were tested inside the test facility (**B**). The photos are used with permission from Lead Healthcare.

Participants were recruited at one of the Lead Healthcare test facilities across the Netherlands, where they sought SARS-CoV-2 testing. Mass testing was conducted at locations referred to as ‘test streets’, since participants could enter by foot, bicycle or car. Participants who entered by car were tested while remaining in their car (see Figure 1A), while those who came by foot or bicycle were tested inside the test facility (see Figure 1B).

On-site testing employed either the PanbioTM COVID-19 antigen rapid test device (Abbott Diagnostic GmbH, Jena, Germany) or the Roche SARS-CoV-2 rapid antigen test (Roche Diagnostics, Basel, Switzerland), depending on availability and participant preference. Previous research revealed no difference in sensitivity of the two tests [8]. All procedures were standardized to maintain consistency and reliability in test administration and analysis, conducted by trained healthcare professionals adhering to rigorous safety and quality protocols. Trained Lead Healthcare personnel analyzed samples, and participants received test results via email within half an hour, indicating either a positive (SARS-CoV-2 detected) or negative (no SARS-CoV-2 detected) outcome. The same email invited participants to complete a brief online survey covering demographics, experienced COVID-19 symptoms on the test day, and mood, health, and quality of life in the week preceding the test.

Demographics details encompassed age (years), sex (male or female), bodyweight (kg), and height (m). At the time of testing, the presence and severity of seventeen COVID-19-related symptoms were assessed, corresponding to the Centers for Disease Control and Prevention (CDC) symptoms listing for the beta variant of SARS-CoV-2 [2]. These symptoms included congestion, runny nose, sore throat, coughing, headache, shivering, fever, shortness of breath, chest pain, fatigue, muscle pain, loss of smell, confusion, difficulty staying awake, blue lips, nausea, and diarrhea. Symptom severity was rated as none (0), mild (1), moderate (2) or severe (3). Overall COVID-19 symptom severity was calculated as the average rating across the 17 symptoms. The number of COVID-19-related symptoms present equaled the sum of symptoms with a score > 0. Participants who reported no COVID-19 symptoms were classified as ‘asymptomatic’, whereas participants who reported one or more COVID-19 symptom were classified as ‘symptomatic’.

Past week’s mood was assessed using single item scales ranging from absent (score 0) to extreme (score 10) [9,10]. Mood items included stress, anxiety, depression, fatigue, hostility, loneliness, happiness, and concentration problems. Overall mental fitness was assessed using a single item scale ranging from very poor (score 0) to excellent (score 10) [11]. Health correlates included overall physical fitness and sleep quality, measured with single item scales ranging from very poor (score 0) to excellent (score 10) [11,12]. Past week’s pain was assessed using single item scales ranging from absent (score 0) to extreme (score 10). Additionally, the 3-item Pain Catastrophizing Scale (PCS) was completed, including items on rumination, magnification, and helplessness [13]. Items were rated from 1 (not at all) to 5 (always). A sum score was computed to represent overall pain catastrophizing. Quality of life was assessed using single item scales ranging from very poor (score 0) to excellent (score 10) [10].

Statistical analysis, performed using SPSS (IBM Corp. Released 2013. IBM SPSS Statistics for Windows, Version 29.0. Armonk, NY, USA: IBM Corp.), involved computing mean and standard deviation (SD) for all variables. Data distributions were checked for normality using the Kolmogorov–Smirnov test and visual inspection, revealing a non-normal distribution. Therefore, differences symptom severity ratings and mood and health scores between those who tested positive and negative for COVID-19 were analyzed using the Independent-Samples Mann-Whitney U Test. Differences were considered statistically significant if *p* < 0.05 (2-tailed). A Bonferroni’s correction was applied to correct for multiple comparisons for COVID-19-related symptoms (17 comparisons, *p* < 0.00294 for significance), mood (9 comparisons, *p* < 0.0055 for significance) and health items (7 comparisons, *p* < 0.0071 for significance). For the other variables, *p* < 0.05 was used as a cut-off for significance. Differences in percentages of individuals that reported COVID-19-related symptoms were analyzed using the N-1 Chi Squared test [14,15], using MedCalc Software Ltd. (comparison of proportions calculator—https://www.medcalc.org/calc/comparison_of_proportions.php (Version 22.017; accessed 16 January 2024). Finally, a logistic regression analysis was used to investigate which symptoms best predict testing positive or negative for SARS-CoV-2. Two models were fitted: (a) Model 1, where all 17 symptoms were predictors of SARS-CoV-2 status (0 = negative, 1 = positive), and (b) Model 2, where only the significant predictors of Model 1 were included as predictors for SARS-CoV-2 status.

## 3. Results

A total of *n* = 88 tested positive and *n* = 837 tested negative for SARS-CoV-2. Among those testing positive, 8% (7 out of 88) were asymptomatic, whereas 20.9% (175 out of 837) of those testing negative reported no COVID-19-related symptoms. The difference in the percentage of individuals who reported no COVID-19-related symptoms between the positive and negative groups was statistically significant (*p* = 0.004).

Overall, individuals testing positive for SARS-CoV-2 reported a significantly greater mean (SD) number of COVID-19-related symptoms compared to those testing negative for SARS-CoV-2: 5.2 (3.2) versus 3.4 (3.0), *p* < 0.001. Additionally, the mean (SD) overall symptom severity score was significantly higher in the positive group compared to the negative group: 0.46 (0.4) versus 0.29 (0.3), *p* < 0.001.

**Figure 2:**
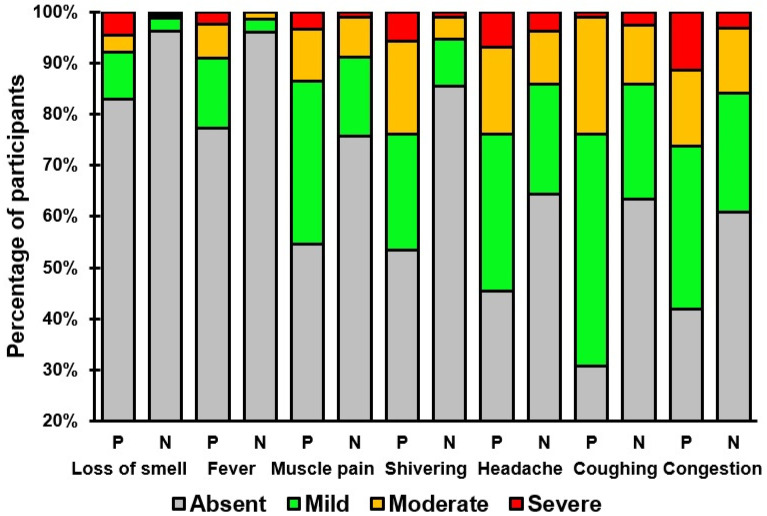
Severity ratings of COVID-19 symptoms that significantly differed between individuals who tested positive (P) or negative (N) for SARS-CoV-2.

Table 2 and Table 3 present an overview of the presence and severity of the individual COVID-19 symptoms in individuals that tested positive or negative for SARS-CoV-2. Symptoms such as coughing, congestion, headache, shivering, muscle pain, fever, and loss of smell were significantly more prevalent and had higher severity scores among individuals testing positive (See Table 3 and Figure 2). No significant differences were found for the common symptoms of fatigue, runny nose, and sore throat, or the infrequently reported symptoms of shortness of breath, diarrhea, difficulty staying awake, nausea, chest pain, confusion, and blue lips.

Mood, health, and quality of life ratings, referring to the week before the SARS-CoV-2 test, are summarized in Table 4. No significant differences were found between individuals that tested positive or negative for SARS-CoV-2, except for significantly higher stress ratings among individuals who tested negative for SARS-CoV-2.

The results of the binary logistic regression analysis are summarized in Table 5. A log-likelihood ratio test showed that including all 17 symptoms to the model did not improve predictive power for SARS-CoV-2 diagnosis, X2(1) = 12.32, *p* = 0.264. Thus, the non-significant predictors from Model 1 were omitted for the interpretation of Model 2. Model 2 had an overall predictive validity of 13.4%.

The symptom severity in this was study was rated using the levels absent (0), mild (1), moderate (2), and severe (3). The analysis revealed that a 1 level increase in the severity of congestion, coughing, shivering, or loss of smell was associated with an increase in the odds of testing positive for SARS-CoV-2 by 1.70, 1.53, 2.77, or 1.88 times, respectively. Alternatively, by taking the inverse of the odd ratios that are less than 1, a 1 level increase in the severity of runny nose, sore throat, or fatigue was associated with an increase in the odds of testing negative for SARS-CoV-2 by 1.51, 1.54, or 1.69 times, respectively.

## 4. Discussion

The current study highlighted that individuals who tested positive for SARS-CoV-2 reported significantly higher frequencies and severity scores of symptoms such as coughing, congestion, headache, shivering, muscle pain, fever, and loss of smell compared to those who tested negative. The logistic regression analysis further indicated that elevated severity levels of congestion, coughing, shivering, or loss of smell were associated with an increased likelihood of testing positive for SARS-CoV-2. Conversely, higher severity levels of runny nose, sore throat, or fatigue were associated with an increased likelihood of testing negative for SARS-CoV-2. Regarding mood and health correlates, no significant differences were observed between individuals testing positive or negative, except for a significantly higher stress score among those who tested negative for SARS-CoV-2.

The absence of reporting any COVID-19 symptoms was not limited to 20.9% of individuals that tested negative for SARS-CoV-2, but also seen among 8% of those who tested positive for SARS-CoV-2. The latter underscores the diverse clinical presentation of SARS-CoV-2 infection and highlights the potential for asymptomatic transmission.

The observation that symptoms such as coughing, congestion, headache, shivering, muscle pain, fever, and loss of smell were more prevalent and more severe among those who tested positive for SARS-CoV-2 aligns with previous research indicating unique symptom clusters in COVID-19 patients [16,17,18,19,20,21,22]. Notably, the association of loss of smell with COVID-19, a symptom less common in other respiratory viruses, has been highlighted as a distinguishing feature in several studies, with a significantly higher prevalence in COVID-19 patients compared to those with other respiratory infections [23,24,25,26,27,28,29]. The prevalence of anosmia in COVID-19 patients is high, with up to 85% of patients reporting subjective symptoms and up to 98% showing objective olfactory dysfunction [29]. The exact mechanism of anosmia in COVID-19 is still being investigated, with potential causes including direct damage to olfactory sensory neurons and impairment of the olfactory perception center in the brain [30].

The logistic regression analysis revealed that increased severity in congestion, coughing, shivering, or loss of smell was associated with a higher likelihood of testing positive for SARS-CoV-2. These findings are consistent with previous work aiming to distinguish the clinical presentations of individuals who tested positive or negative for SARS-CoV-2 [31,32]. Conversely, symptoms such as a runny nose and sore throat, often associated with common colds, were linked to negative test results, underscoring the symptomatic overlap between COVID-19 and other respiratory infections [33].

While these findings might suggest that the presence and severity of loss of smell could be used as a guidance in the decision whether or not to test for SARS-CoV-2, this is unlikely to be a successful strategy—that is, COVID-19 is characterized by a wide variety of other symptoms that can occur in different combinations and severity levels, often without the presence of loss of smell. Moreover, a substantial minority of individuals who test positive for SARS-CoV-2 was asymptomatic (8% in the current study).

This study had some limitations. First, it did not investigate the motives for testing, which could have influenced the high number of negative tests. For example, in the Netherlands, negative SARS-CoV-2 tests were required for entry into social venues, such as bars and restaurants, leading to a substantial number of asymptomatic individuals opting for SARS-CoV-2 testing. Alternatively, fear of COVID-19 may have influenced the decision of individuals to get tested, as indicated by significantly higher stress scores among those who testing negative. Despite the fact that the symptom presence and severity of this group was significantly lower compared to those who tested positive for SARS-CoV-2, it can be speculated that fear of COVID-19 may have been an important reason for these participants to get tested. To enable better interpretation of the study findings, future studies should therefore assess motives for testing and fear of COVID-19. Second, it is important to highlight that the current study evaluated symptoms of the beta variant of SARS-CoV-2. For other variants, different symptoms may be more dominant in the clinical presentation of COVID-19. Therefore, this study should be replicated in future for other variants of SARS-CoV-2. Third, the severity level of COVID-19 symptoms was scored as absent, mild, moderate, or severe. However, no guidelines were provided to participants on how to interpret these levels. Therefore, severity levels may have been interpreted differently by individual participants. It is therefore recommended for future research that scoring guidelines or descriptions of the severity levels are provided along with the questions. Fourth, whereas COVID-19 symptoms were reported in real time, the mood and health correlates were reported retrospectively for the past week. As such, recall bias may have influenced the mood and health assessments. In future studies, this can be ruled out by using a prospective study design with real time assessments for all assessed variables. Lastly, the use of rapid antigen tests, while providing quick results, may entail a margin for error, with the possibility of false positives and false negatives [34,35,36]. In future studies, it is recommended that more precise molecular tests, such as polymerase chain reaction (PCR) and other nucleic acid amplification tests (NAATs) tests are used.

## 5. Conclusions

Increased severity levels of congestion, coughing, shivering, or loss of smell were associated with an increase in the odds of testing positive for SARS-CoV-2, while higher severity levels of runny nose, sore throat, or fatigue were associated with an increased likelihood of testing negative for SARS-CoV-2.

## Figures and Tables

**Table 1:** A comparison between common cold, flu, and COVID-19 symptoms.

Symptoms	Common Cold	Flu	COVID-19
Coughing	√	√	√
Runny nose	√	√	√
Sore throat	√	√	√
Headache	√	√	√
Muscle pain	√	√	√
Fever (a body temperature ≥ 38 Celsius)	√	√	√
Shivering		√	√
Diarrhea		√	√
Fatigue		√	√
Congestion	√		√
Sneezing	√		
Loss of smell			√
Shortness of breath			√
Difficulty staying awake			√
Nausea			√
Chest pain			√
Confusion			√
Blue lips			√

Symptoms of common cold, flu, and COVID-19 (beta variant), as reported by the US Centers for Disease Control and Prevention (CDC) (available from: www.cdc.gov (accessed on 24 June 2024), references [2,3,4]). √ = symptom present. Abbreviation: COVID-19 = 2019 coronavirus disease.

**Table 2:** Presence and severity of COVID-19 symptoms.

	COVID-19 Symptoms—Presence			COVID-19 Symptoms—Severity		
Test Group	Negative	Positive		Negative	Positive	
Symptoms	Reported by (%)	Reported by (%)	*p*-Value	Mean (SD)	Mean (SD)	*p*-Value
Congestion	39.2	58.0	<0.001 *	0.58 (0.8)	0.95 (1.0)	<0.001 *
Coughing	36.6	69.3	<0.001 *	0.53 (0.8)	0.94 (0.8)	<0.001 *
Headache	35.7	54.5	<0.001 *	0.54 (0.8)	0.85 (0.9)	<0.001 *
Shivering	14.6	46.6	<0.001 *	0.21 (0.6)	0.76 (0.9)	<0.001 *
Muscle pain	24.3	45.5	<0.001 *	0.34 (0.7)	0.63 (0.8)	<0.001 *
Fever	3.9	22.7	<0.001 *	0.06 (0.3)	0.34 (0.7)	<0.001 *
Loss of smell	3.7	17.0	<0.001 *	0.06 (0.3)	0.30 (0.7)	<0.001 *
Fatigue	50.9	52.3	0.803	0.77 (0.9)	0.85 (0.9)	0.493
Runny nose	38.4	43.2	0.380	0.57 (0.8)	0.63 (0.8)	0.405
Sore throat	26.9	34.1	0.151	0.57 (0.8)	0.53 (0.8)	0.658
Shortness of breath	13.6	22.7	0.021	0.17 (0.4)	0.27 (0.5)	0.022
Difficulty staying awake	11.1	12.5	0.693	0.15 (0.5)	0.19 (0.5)	0.628
Diarrhea	15.9	15.9	1.000	0.21 (0.5)	0.17 (0.4)	0.915
Nausea	8.4	11.4	0.342	0.11 (0.4)	0.14 (0.4)	0.347
Chest pain	8.5	9.1	0.848	0.10 (0.4)	0.10 (0.3)	0.860
Confusion	2.7	5.7	0.116	0.03 (0.2)	0.06 (0.2)	0.130
Blue lips	0.6	1.1	0.578	0.01 (0.1)	0.02 (0.2)	0.544

Mean (SD) scores for each symptom are presented. Differences between the groups who tested positive and negative for SARS-CoV-2 are considered statistically significant if *p* < 0.00294 (after Bonferroni’s correction for multiple comparisons), and indicated by *. Abbreviation: COVID-19 = 2019 coronavirus disease.

**Table 3:** COVID-19 symptom presence according to severity.

	Presence of Symptoms Associated with COVID-19 According to Severity							
	Tested Negative for COVID-19				Tested Positive for COVID-19			
Symptom Severity	Absent	Mild	Moderate	Severe	Absent	Mild	Moderate	Severe
Congestion	60.8	23.3	12.7	3.2	30.7 *	45.5	22.7	1.1 *
Coughing	63.4	22.5	11.5	2.6	42.0 *	31.8 *	14.8 *	11.4
Headache	64.3	21.5	10.5	3.7	45.5 *	30.7	17.0	6.8
Shivering	85.4	9.3	4.2	1.1	47.7 *	23.9 *	23.9 *	4.5 *
Muscle pain	75.7	15.4	7.8	1.1	53.4 *	22.7 *	18.2	5.7
Fever	96.1	2.4	1.4	0.1	54.5 *	31.8 *	10.2 *	3.4 *
Loss of smell	96.3	2.5	0.6	0.6	56.8 *	26.1 *	14.8	2.3 *
Fatigue	49.1	29.2	17.0	4.8	65.9	18.2	12.5	3.4
Runny nose	61.6	23.7	10.8	3.9	77.3	13.6	6.8	2.3
Sore throat	63.1	20.8	12.7	3.5	77.3	18.2	4.5	0.0
Shortness of breath	86.4	10.8	2.7	0.1	83.0	9.1	3.4	4.5
Diarrhea	84.1	11.9	3.0	1.0	84.1	14.8	1.1	0.0
Difficulty staying awake	88.9	8.0	2.5	0.6	87.5	5.7	6.8	0.0
Nausea	91.6	6.5	1.6	0.4	88.6	9.1	2.3	0.0
Chest pain	91.5	6.8	1.6	0.1	90.9	8.0	1.1	0.0
Confusion	97.3	2.3	0.5	0.0	94.3	5.7	0.0	0.0
Blue lips	99.4	0.6	0.0	0.0	98.9	0.0	1.1	0.0

Percentage of individuals (%) are shown that reported symptoms being either absent, mild, moderate or severe. Significant differences (*p* < 0.00294, applying a Bonferroni’s correction for multiple comparisons) between the groups who tested positive or negative for SARS-CoV-2 are indicated by *. Abbreviation: COVID-19 = 2019 coronavirus disease.

**Table 4:** Mood, health, and quality of life in the week before the SARS-CoV-2 test.

Test Group	Tested Negative for COVID-19	Tested Positive for COVID-19	*p*-Value
Mood			
Stress	2.4 (2.9)	1.5 (2.4)	0.002 *
Anxiety	1.2 (2.2)	0.8 (1.9)	0.069
Depression	0.8 (1.7)	0.6 (1.4)	0.224
Fatigue	3.6 (2.9)	2.8 (2.7)	0.012
Hostility	0.6 (1.6)	0.3 (0.8)	0.652
Loneliness	1.0 (2.0)	0.6 (1.4)	0.202
Happiness	6.6 (2.1)	6.9 (2.0)	0.345
Concentration problems	2.1 (2.6)	1.7 (2.6)	0.058
Mental fitness	6.9 (2.1)	7.0 (2.4)	0.487
Health			
Physical fitness	6.3 (2.2)	6.4 (2.3)	0.539
Pain	1.3 (2.0)	1.2 (2.3)	0.245
Pain catastrophizing	4.7 (1.9)	4.5 (1.9)	0.411
PCS—Rumination	1.8 (0.8)	1.7 (0.7)	0.263
PCS—Magnification	1.4 (0.7)	1.3 (0.6)	0.723
PCS—Helplessness	1.5 (0.8)	1.5 (0.9)	0.675
Sleep quality	6.9 (1.9)	7.4 (1.9)	0.028
Quality of life			
Quality of life	7.6 (1.5)	7.8 (1.9)	0.107

Mean (SD) scores are presented. Differences between the groups who tested positive and negative for SARS-CoV-2 are considered statistically significant if *p* < 0.05 for quality of life, and *p* < 0.0055 for mood items and *p* < 0.0071 for health items (after Bonferroni’s correction for multiple comparisons). Statistical significance is indicated by *. Abbreviations: PCS = pain catastrophizing scale, COVID-19 = 2019 coronavirus disease.

**Table 5:** Results of the binary logistic regression analysis.

	Model 1			Model 2		
Predictors	Odds Ratios	95% CI	*p*	Odds Ratios	95% CI	*p*
Congestion	1.61	1.14–2.25	0.006 *	1.70	1.22–2.36	0.001 *
Runny nose	0.63	0.43–0.89	0.012 *	0.66	0.47–0.93	0.021 *
Sore throat	0.67	0.47–0.94	0.025 *	0.65	0.46–0.89	0.010 *
Coughing	1.53	1.09–2.13	0.013 *	1.53	1.11–2.11	0.009 *
Headache	1.19	0.84–1.68	0.320			
Shivering	2.72	1.77–4.23	<0.001 *	2.77	1.98–3.92	<0.001 *
Fever	1.46	0.85–2.54	0.177			
Shortness of breath	1.17	0.64–2.09	0.600			
Chest pain	0.51	0.20–1.14	0.124			
Fatigue	0.61	0.41–0.88	0.010 *	0.59	0.42–0.82	0.002 *
Muscle pain	1.08	0.72–1.60	0.716			
Loss of smell	1.94	1.19–3.17	0.008 *	1.88	1.22–2.91	0.004 *
Confusion	1.23	0.35–3.55	0.724			
Difficulty staying awake	1.03	0.57–1.77	0.906			
Blue lips	2.02	0.31–10.41	0.395			
Nausea	0.50	0.21–1.12	0.110			
Diarrhea	0.77	0.40–1.34	0.381			
Observations	925			925		
R^2^ Tjur	0.157			0.134		

Significant *p*-values (*p* < 0.05) are indicated by *. Abbreviation: CI = confidence interval.

## Data Availability

The data are available from the corresponding author upon reasonable request.
